# Assessment of noninvasive positive pressure ventilation in healthy young volunteers using salivary stress biomarkers

**DOI:** 10.2144/fsoa-2019-0150

**Published:** 2020-02-24

**Authors:** Yuri Fueda, Fuka Matsuda, Takuya Kataoka

**Affiliations:** 1 Department of Medical Engineering, Faculty of Health Sciences, Morinomiya University of Medical Sciences, Osaka, Japan; 2 Department of Medical Engineering, Faculty of Health Sciences, Himeji Dokkyo University, Himeji, Hyogo, Japan

**Keywords:** amylase, chromogranin A, humidity, leak volume, mask, noninvasive ventilation, saliva

## Abstract

**Aim:**

We performed a stress assessment of noninvasive positive pressure ventilation (NPPV) using the salivary biomarkers chromogranin A.

**Materials & methods:**

Twenty healthy volunteers were subjected to NPPV for 30 min. We collected saliva samples before and after NPPV and evaluated chromogranin A.

**Results:**

We collected saliva samples from 13 volunteers for enzyme measurement. Of the 13 volunteers, 11 showed elevated chromogranin A levels, which were significantly higher after NPPV than before NPPV (p < 0.01). The chromogranin A increase group displayed significantly increased leak flow and reduced respiratory rate and absolute humidity compared with the chromogranin A reduction group.

**Conclusion:**

The increase of leak volume might be a stress factor in patients receiving NPPV.

## Lay abstract

Salivary biomarkers are attractive as they are noninvasive and convenient. Noninvasive positive pressure ventilation (NPPV), the delivery of mechanical ventilation to those with respiratory failure, can have complications resulting in stress to the patient. Here, we evaluated stress-related salivary biomarkers during NPPV in healthy individuals. Our results suggest that NPPV might increase stress levels and that humidity and leak volume affected stress.

The number of studies assessing stress using salivary biomarkers has been increasing because biomarker assessment is noninvasive, occasional and convenient [1]. Stress increases the activity of the sympathetic–adrenal nervous system, which is involved in the release of catecholamines from the adrenal medulla and in the activation of the hypothalamus–pituitary–adrenal axis [2]. The activation of these two responses is associated with changes in salivary biomarkers, such as cortisol, amylase and chromogranin A. For example, salivary amylase levels have been reported to significantly increase due to the calculation load [3]. Hill *et al.* reported that exercise increases plasma cortisol levels [4]. In addition, Filaire *et al.* demonstrated that public speaking reportedly increased salivary amylase activity and cortisol levels, but it did not cause any changes in chromogranin A levels [5]. Cortisol is an endocrine hormone that is secreted from the adrenal cortex via the hypothalamus–pituitary–adrenal axis in response to physical stress [1]. Amylase is a salivary enzyme and chromogranin A is an acidic glucoprotein. These two biological markers, which can also serve as psychological markers, are produced by catecholamines released from the adrenal medulla before the activation of the sympathetic nervous system [6–8]. Because they are also distributed in the submandibular gland, they are secreted into the saliva by sympathetic nervous stimuli (Figure 1).

**Figure 1.**
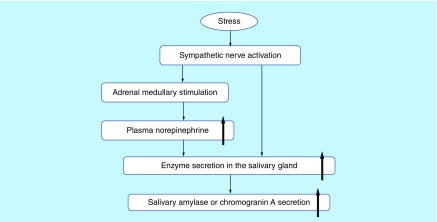
Mechanism of salivary enzyme secretion under stress. Levels of chromogranin A and amylase, as psychological stress biomarkers, increase in two pathways, which are involved in the activity of the sympathetic–adrenal nervous system as well as the activation of the salivary gland via direct stimulation of the sympathetic nervous [1,2].

Noninvasive positive pressure ventilation (NPPV) has an open-circuit design. The delivered flow of gas increases to compensate for the leak volume in the system [9–11]. However, a high flow causes complications, such as ear and sinus pain, gastric insufflation, ulcers, upper airway dryness and eye irritation [12,13]. The present study performed a stress assessment of NPPV using the salivary biomarkers chromogranin A and amylase.

## Materials & methods

### Study setting

This study was conducted at Himeji Dokkyo University from 2015 to 2016. The study protocol was reviewed and approved by the Himeji Dokkyo University Ethics Committee (number: 15-03) and all participants provided verbal consent.

### Volunteers

We conducted breathing function tests on 20 college students (males: 15; females: 5 [Table 1]). These volunteers were healthy with no systemic illness and no ongoing medical treatment. They had forced vital capacity of 80% or more and forced expiratory volume of 70% or more in breathing function test. We excluded students with breathing function tests outside of the normal range and medical history. We verbally explained the purpose of the experiment to obtain consent before starting this study.

**Table 1.** Volunteer breathing function test results.

Male/female, no.	15/5
Mean age (year)	20.95 ± 0.83
Height (cm)	165.35 ± 9.56
Weight (kg)	61.87 ± 19.79
FVC (%)	103.45 ± 14.94
FEV 1.0%	103.04 ± 9.52

Values are the median ± standard deviation.

FEV: Forced expiratory volume; FVC: Forced vital capacity.

### Equipment

NPPV was provided using a bi-level positive airway pressure ventilator (BiPAP vision, Philips Respironics, PA, USA). The ventilator circuits comprised a standard 180-cm length of smooth-bore tubing, a heated humidifier (PMH1000, Pacific Medico, Tokyo, Japan) and an exhalation port. The interface comprised an optimal oronasal mask (Teijin, Tokyo, Japan) pressed against the volunteer’s face. The ventilator settings were as follows: spontaneous and timed mode for 30 min, with an inspiratory positive airway pressure of 10 cm H_2_O, an expiratory positive airway pressure of 5 cm H_2_O, a respiratory rate of 12 breaths/min and an inspired oxygen (FiO_2_) rate of 21%. We confirmed the exhaled ventilation volume, leak volume and respiratory rate on the ventilator monitor.

The following two different temperatures of a heated humidifier were assessed: medium and maximum degrees. The absolute humidity of the inspired gas during NPPV was measured using a temperature and humidity monitor (MAPHY+, Skynet, Osaka, Japan).

A pulse oximeter was attached to the volunteer’s figure to measure heart rate and blood oxygen levels (SpO2). A multi-monitoring system (DS-7300, Fukuda Denshi, Tokyo, Japan) was used to measure heart rate and SpO_2_.

### Sample collection & salivary biomarker analysis

We used Salivette™ cotton swabs (Sarstedt, Tokyo, Japan) to collect saliva before and after NPPV. The Salivette cotton swab was placed in the right or left cheek for 90 s to absorb saliva before NPPV. In the case of the end of NPPV, cotton swab was immediately placed in the same cheek as before NPPV for 90 s. We instructed volunteers to let saliva absorb with cotton swab. The samples were centrifuged for 2 min at 3000 g at 4°C and the protein content was quantified with the BCA Protein Assay Kit (Takara Bio Inc., Shiga, Japan) after collection. The protein was standardized to a concentration of 50 μg/ml and the samples were stored at -80°C to minimize protein activity until the salivary biomarkers were measured.

Salivary amylase activity was measured using a portable salivary amylase monitor (Nipro Co., Osaka, Japan) [14]. In general, a test strip was placed directly under the volunteer’s tongue for approximately 30 s to absorb saliva and was then entered into the monitor. Salivary amylase finally appeared after 60 s [4]. However, the salivary amylase was not measured because it was not absorbed in the saliva in the cotton swab. To overcome this complication, we measured precisely 28 μl of saliva according to the method by Yamaguchi *et al.* and dropped the samples on the tip [3].

Salivary chromogranin A levels were determined using enzyme immunoassay kits (YK070 Human Chromogranin A EIA kit, Yanaihara Institute, Inc., Shizuoka, Japan).

### Statistical analysis

The results were described as the means ± standard deviation. The differences were analyzed using paired t-tests and Wilcoxon signed rank test. Comparisons between groups were analyzed using Student’s t-test. All statistical differences were considered significant with a p-value of <0.05. All statistical analyses were performed using Statcel2 (OMS Ltd, Saitama, Japan).

## Results

### Assessment of salivary biomarkers

We were able to collect a sufficient amount of saliva from 13 of the 20 volunteers to assess salivary biomarkers. To compare before and after NPPV, we used samples from volunteers collected before and after NPPV and others were excluded.

**Figure 2.**
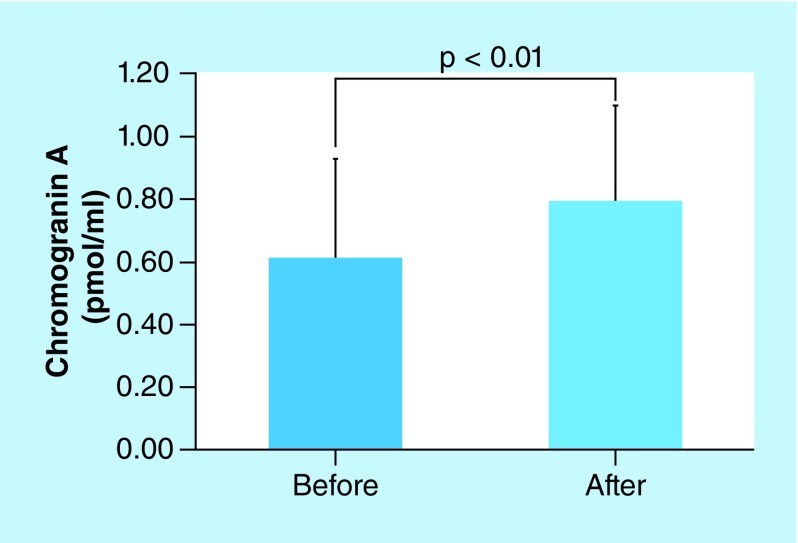
Chromogranin A before and after noninvasive positive pressure ventilation. The average levels of chromogranin A before and after NPPV were 0.612 ± 0.314 and 0.793 ± 0.304 pmol/mL, respectively. Chromogranin A levels were significantly influenced by NPPV. NPPV: Noninvasive positive pressure ventilation.

**Figure 3.**
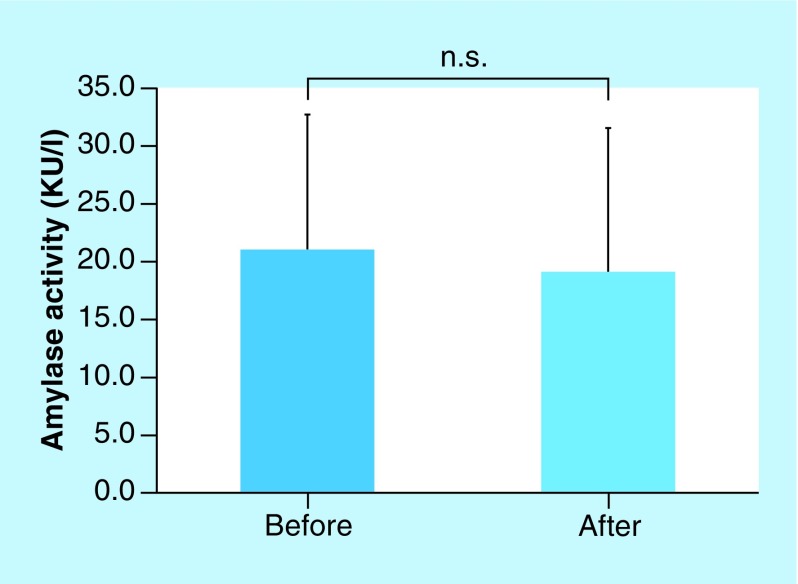
Amylase activity before and after noninvasive positive pressure ventilation. The average amylase activities before and after NPPV were 21.07 ± 11.7 and 19.13 ± 12.4 KU/l, respectively. There was no difference in amylase activity between before and after NPPV (n.s.). NPPV: Noninvasive positive pressure ventilation; n.s: Not significant.

Chromogranin A levels were elevated in 11 of the 13 volunteers. The average levels of chromogranin A before and after NPPV were 0.612 and 0.793 pmol/ml, respectively. Chromogranin A levels were significantly influenced by NPPV (p < 0.01, Figure 2), whereas amylase activity was not responsive to NPPV. The average amylase activities before and after NPPV were 21.07 and 19.13 KU/l, respectively (Figure 3). Amylase activities increased in five volunteers and decreased in seven volunteers. One volunteer did not display any changes in amylase activity.

### Association between chromogranin A & NPPV parameters

**Figure 4.**
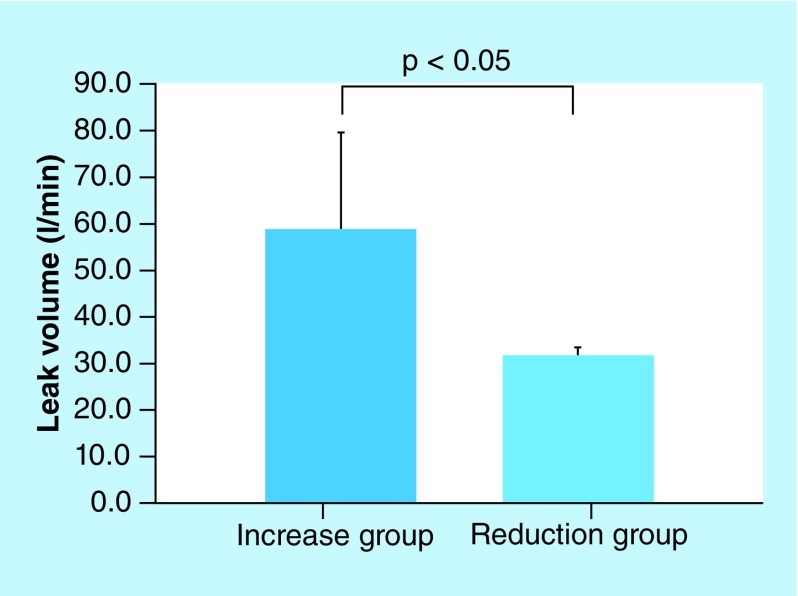
Association between chromogranin A and leak volume. Leak volumes in the chromogranin A increase group and the chromogranin A reduction group were 58.64 ± 41.37 and 31.5 ± 3.38 l/min, respectively. The difference in leak volumes between the increase and reduction groups was statistically significant (p < 0.05).

**Figure 5.**
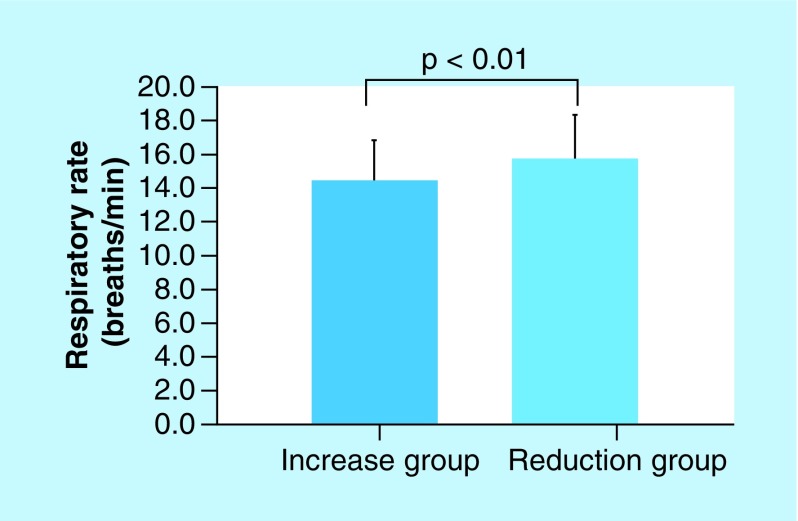
Association between chromogranin A and respiratory rate. Respiratory rates in the chromogranin A increase group and the chromogranin A reduction group were 14.72 ± 2.25 and 17.95 ± 2.65 breaths/min, respectively. The difference in respiratory rates between the increase and reduction groups was statistically significant (p < 0.05).

**Figure 6.**
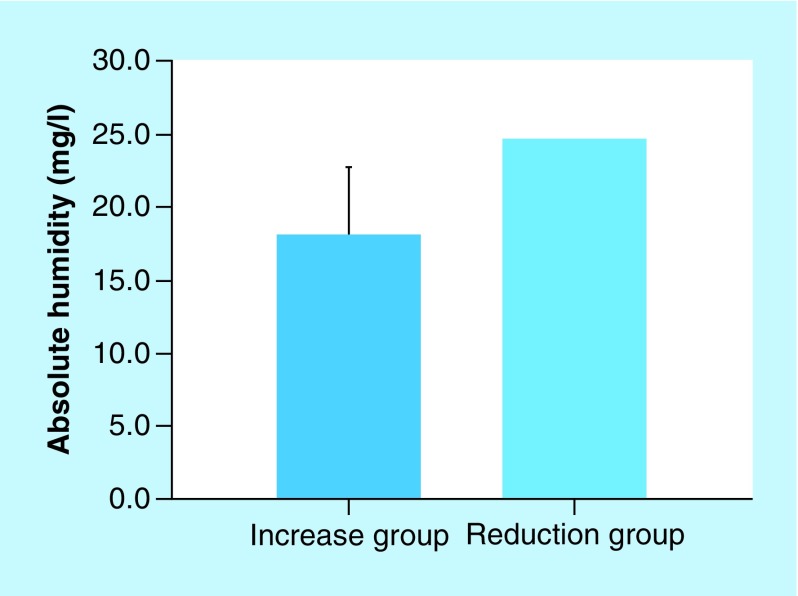
Association between chromogranin A and absolute humidity. Absolute humidity was higher in the reduction group (24.53 mg/l) than in the increase group (18.08 ± 4.63 mg/l). However, the difference in absolute humidity between the increase and reduction groups could not be analyzed statistically since there were not enough samples in the reduction group.

The leak volumes in the chromogranin A increase group and the chromogranin A reduction group were 58.64 ± 41.37 and 31.5 ± 3.38 l/min, respectively. The respiratory rates in the chromogranin A increase group and the chromogranin A reduction group were 14.72 ± 2.25 and 17.95 ± 2.65 breaths/min, respectively. The differences between the increase and reduction groups were statistically significant (p < 0.05; Figures 4 & 5). In addition, the absolute humidity was higher in the reduction group than in the increase group. However, the difference in absolute humidity between the increase and reduction groups could not be statistically analyzed because there were not enough samples in the reduction group (Figure 6). The other parameters are described in Table 2. No other parameters affected chromogranin A levels.

**Table 2.** Relationship between chromogranin A and parameters in noninvasive positive pressure ventilation (n = 13).

Items	Increase group	Reduction group	p-value
Increase group exhaled volume (ml)	781.71 ± 466.94	598.35 ± 209.56	n.s.
SpO_2_ (%)	98.78 ± 0.98	99.75 ± 0.44	n.s.
Heart rate (rate/min)	79.85 ± 11.46	74.05 ± 17.4	n.s.

Values are the median ± standard deviation.

n.s.: Not significant.

### Association between chromogranin A & inspired gas humidity

**Figure 7.**
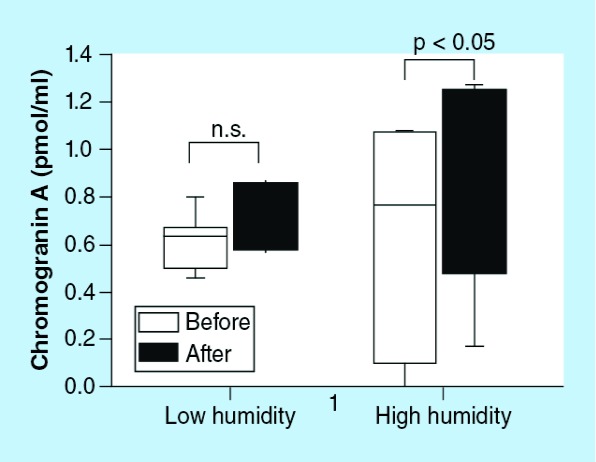
Association between chromogranin A and inspired gas humidity. Absolute humidity at the medium and maximum temperatures was 14.64 ± 3.02 and 22.21 ± 1.75 mg/ml, respectively. Chromogranin A levels were not responsive to low inspired gas humidity, whereas chromogranin A levels were significantly influenced by high inspired gas humidity (p < 0.05). n.s.: Not significant.

The absolute humidity at the medium and maximum temperatures was 14.6 ± 3.02 and 22.20 ± 1.75 mg/ml, respectively. Chromogranin A levels were not responsive to low inspired gas humidity, whereas chromogranin A levels were significantly influenced by high inspired gas humidity (p < 0.05; Figure 7).

## Discussion

NPPV has been shown to be effective for patients with respiratory failure, heart failure and sleep apnea syndrome [15–17]. NPPV can be immediately applied to patients to supply gas via nasal or full-face masks without undergoing tracheal intubation. However, the patients can easily remove the masks, so they may do so and not receive assistance with respiration if NPPV were a stress factor. NPPV provides pressure-controlled ventilation by changing the flow volume to compensate for leaks between the face and mask. A large gap between the face and mask may result in redness and ulcer development. If the flow volume increases due to increasing leak volume, the patient may experience discomfort. Therefore, cooperation between the patients and medical staff is essential. Before starting NPPV, we must explain its risks and benefits to patients and families.

In this study, we used the salivary biomarkers chromogranin A and amylase to assess stress. Chromogranin A is widely distributed in endocrine cells and the nervous system, particularly in the adrenal medulla and pituitary [1,2]. Chromogranin A is also produced by the submandibular gland and has been shown to be secreted into saliva by autonomic nerve stimulation [6]. Chromogranin A has a higher sensitivity to psychological stress than to exercise stress [18]. Nakane *et al.* reported that chromogranin A levels were elevated immediately before oral presentations and decreased immediately after the presentation for 15 min, whereas salivary chromogranin A levels may not be significantly influenced by 50 min of driving. We confirmed that the saliva collection time was optimal in this study. In another study, elevated norepinephrine levels were associated with elevated salivary amylase levels [19]. Norepinephrine activity is controlled by the sympathetic adrenal medullary system. In addition, amylase reacts promptly because it is directly acted upon by the sympathetic nervous system [1]. We assumed that our results were able to assess stress levels from saliva collected 2 min after NPPV completion.

The average chromogranin A levels were significantly different before and after NPPV; however, amylase activity did not change. Similar to our results, other reports have shown that the chromogranin A changes were inconsistent with those of amylase. Guilhem *et al.* reported that the chromogranin A levels and amylase activity showed different changes in swimmers than in other competitive sports [20]. Shinjo *et al.* reported that chromogranin A level decreased and amylase activity increased in the calculation load [21]. They showed that it was necessary to assess individual changes. Murakami *et al.* associated amylase activity with feelings such as comfort or anxiety. High-level volunteers according to the State-Trail Anxiety Inventory, which was used as an index of the state of anxiety, showed higher amylase activity than low-level volunteers [22]. Therefore, chromogranin A was used to evaluate psychological stress in this study. We assumed that amylase activity can be used to distinguish between the psychological stress levels of comfort and anxiety.

To the best of our knowledge, there have been no previous reports assessing stress in NPPV. Takashima *et al.* investigated the stressful experiences of ICU patients who were on artificial respirators for 12 h or longer [23]. Samuelson *et al.* reported ICU patients who underwent mechanical ventilation for more than 24 h had unpleasant and pleasant memories [24]. These authors suggested that the stressful experience of the ICU stay was associated with tracheal intubation. Tracheal intubation induces psychological stress, which is triggered by conversation difficulties and the physical restraint of movement. NPPV can assist patient breath without undergoing tracheal intubation. Therefore, we presumed that the stress factors in NPPV and mechanical ventilation will vary. The chromogranin A increase group displayed a significantly increased leak flow and reduced absolute humidity compared with the chromogranin A reduction group. Oto *et al.* associated leak flow with absolute humidity [25]. Leak flow increases the gas flow delivered by NPPV, which supplies an increased proportion of dry fresh gas for mixing with expired gas. Another report also showed that relative humidity decreased with increasing gas flow delivered by NPPV [26]. Those authors suggested that a high flow volume released warm, humid air through an exhalation port. We postulated that the chromogranin A reduction group experienced tension and anxiety before NPPV. The group with decreased chromogranin A levels had become accustomed to receiving NPPV for 30 min.

Presently, it is not essential to use a heated humidifier in the NPPV system, according to respiratory guidelines [27]. NPPV delivers inspired gas through the upper airway, which provides 75% of the heat and moisture supplied to the alveoli. However, a heated humidifier is often used in NPPV to prevent problems such as dryness and throat congestion. We evaluated psychological stress with low and high levels of absolute humidity. The two absolute humidity levels evaluated were not significantly different. Therefore, we presumed that an absolute humidity of more than 15 mg/l is necessary. Wiest *et al.* reported that an absolute humidity of less than 10 mg/l was associated with upper airway dryness in continuous positive airway pressure (CPAP) users [28]. By contrast, 50% of NPPV patients experienced severe oral dryness regardless of the absolute humidity level when it was more than 30 mg/l on average [25]. The patients suffered from acute respiratory failure and elicited more oral dryness, resulting in higher respiratory rates, increased mouth breathing and excessive immune responses compared with healthy volunteers. On the basis of our study, an absolute humidity level more than 15 mg/l is desirable. Nevertheless, we may need to adjust absolute humidity if the patient’s condition is poor.

We evaluated psychological stress by focusing on pressure and delivered flow volume in NPPV. Previous reports have shown that redness and ulcers are caused, in part, by the strong contact between the face and mask such as on the forehead, nasal bridge and cheeks [12,13]. The incidence of pressure ulcers was higher with a nasal mask than with a full-face mask.

We suppose that the mask itself is also a stress factor. There are several types of masks, such as nasal-oral, full-face and helmet [29]. It is important, but challenging, for medical workers to control masks according to the patient’s face size. Therefore, the psychological stress associated with many masks must be examined.

## Conclusion

NPPV led to the elevation of psychological stress biomarker levels in healthy individuals. In addition, salivary chromogranin A levels were a more sensitive stress biomarker than amylase levels. The tested stress biomarkers were affected by change in absolute humidity. These results indicate that decreased absolute humidity increases the inspiratory flow depending on the leak volume. We propose that leak volume can serve as a psychological stress factor during NPPV in healthy individuals.

## Future perspective

In this study, volunteers were young and healthy. However, we need to understand the stress status of patients undergoing respiratory induction. At present, we have constructed a pathological model and evaluated stress. Stress assessment surveys have been conducted in additional experiments on stress evaluation using questionnaire and fingertip pulse waves. If we can construct an experimental method, we will seek further patients and collaboration with other institutions in our proceeding study.

**Summary points**

- Saliva biomarkers were used for assessment of stress caused by noninvasive positive pressure ventilation.
- Saliva samples could be noninvasively collected.
- To investigate the cause of the stress, leak flow, the inspiratory flow and absolute humidity were measured.
- Of the 13 volunteers, 11 showed elevated chromogranin A levels.
- Chromogranin A is more sensitive than amylase.
- The chromogranin A increase group displayed significantly increased leak flow and reduced respiratory rate and absolute humidity compared with the chromogranin A reduction group.
- It was important to control leak flow during noninvasive positive pressure ventilation.
- Stress caused by the use of other interfaces and pathological models would also need to be assessed.
